# P-1118. Clinician knowledge, attitudes, and practices around empiric vancomycin use in the pediatric intensive care unit

**DOI:** 10.1093/ofid/ofae631.1305

**Published:** 2025-01-29

**Authors:** Kathleen Chiotos, Jeffrey S Gerber, Preeti Jaggi, Evan E Facer, Matthew I Goldsmith, Jason G Newland, Rebecca G Same, Pranita Tamma, Brandi M Muller, Didien Meyahnwi, Ebbing Lautenbach, Julia E Szymczak

**Affiliations:** Children's Hospital of Philadelphia, Philadelphia, PA; Children's Hospital of Philadelphia, Philadelphia, PA; Emory University, Atlanta, GA; St. Louis Children's Hospital, St. Louis, Missouri; St. Louis Children's Hospital, St. Louis, Missouri; Washington University in St. Louis School of Medicine, St. Louis, Missouri; Children's Hospital of Philadelphia, Philadelphia, PA; Johns Hopkins School of Medicine, Baltimore, MD; University of Utah, Haddon Township, New Jersey; Children's Hospital of Philadelphia, Philadelphia, PA; University of Pennsylvania, Philadelphia, Pennsylvania; University of Utah School of Medicine, Salt Lake City, Utah

## Abstract

**Background:**

Overuse of empiric vancomycin is common in pediatric intensive care units (PICU) despite a low prevalence of infections requiring vancomycin. Determinants of excess vancomycin prescribing are unknown. Our objective was to evaluate PICU clinician knowledge, attitudes, and practices regarding empiric vancomycin.

**Table 1.**
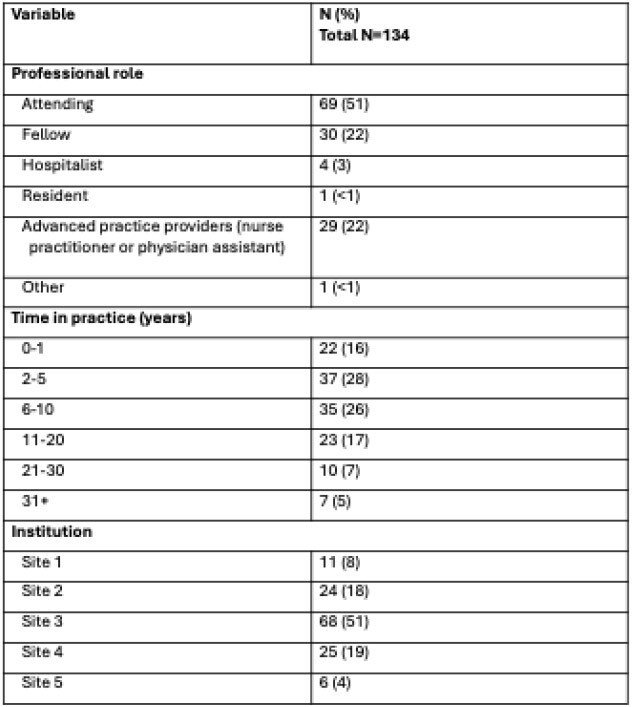
Demographic variables of survey respondents

**Methods:**

We conducted a cross-sectional survey of PICU attendings, fellows, residents, and advanced practice providers in five tertiary care PICUs. The survey was distributed by email in August-September 2023. Survey items included Likert scale (1-5), ranking, and multiple-choice responses. Agree/Strongly Agree and Disagree/Strongly Disagree were collapsed to Agree and Disagree for analysis. Data were analyzed using descriptive statistics.

**Figure 1.**
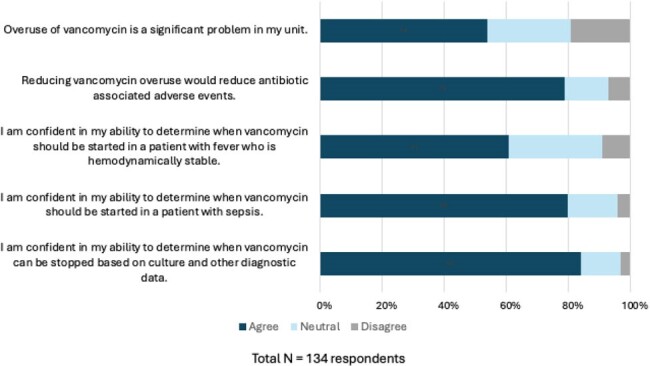
Clinician attitudes about vancomycin use

**Results:**

A total of 134 of 297 eligible clinicians (45%) answered the survey (Table 1). Knowledge of the spectrum of activity of vancomycin was low with only 14 of 117 (12%) respondents answering all questions correctly. Just over half of clinicians agreed that vancomycin overuse was a significant problem (72/134, 54%); this proportion varied from 0% to 65% across sites. Most clinicians (105/133, 79%) agreed that reducing vancomycin overuse would reduce antibiotic-associated adverse events. Clinicians had greater confidence in determining when vancomycin should be given to a patient with sepsis as compared to a hemodynamically stable patient with a fever (107/134, 80% vs 80/131, 61%). Respondents were most confident in determining when vancomycin could be stopped (113/134, 84% agreed) (Figure 1). Illness severity was the factor influencing the decision to start vancomycin ranked highest by the most respondents (55%), followed by suspected source of infection (19%), followed by patient history of methicillin-resistant Staphylococcus aureus (MRSA) (18%); local prevalence of MRSA was ranked highest least often (4%) (Figure 2).

**Figure 2.**
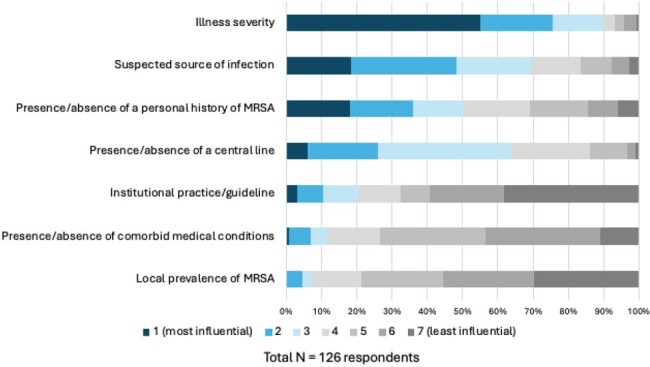
Factors influencing clinician decisions to administer empiric vancomycin

Respondents ranked each possible factor 1-7 with 1 being the most influential factor in choosing to give vancomycin and 7 being the least influential. The blue shading reflects more influential factors and the gray shading reflects less influential factors.

**Conclusion:**

Most PICU clinicians in this multi-site study felt confident about when to stop or start vancomycin, though many misunderstood the spectrum of vancomycin activity. Focusing stewardship efforts on improving clinician knowledge and supporting decision making, particularly for patients without sepsis, may address these barriers.

**Disclosures:**

**Jason G. Newland, MD, MEd**, Moderna: Grant/Research Support|Pfizer: Grant/Research Support

